# Inter‐observer variability in library plan selection on iterative CBCT and synthetic CT images of cervical cancer patients

**DOI:** 10.1002/acm2.14170

**Published:** 2023-10-03

**Authors:** Yvonne J. M. de Hond, Paul. M. A. van Haaren, An‐Sofie E. Verrijssen, Rob H. N. Tijssen, Coen W. Hurkmans

**Affiliations:** ^1^ Department of Radiation Oncology Catharina Hospital Eindhoven Eindhoven The Netherlands

**Keywords:** CBCT, cervix, inter‐observer variability, iterative reconstruction, library of plans, plan‐of‐the‐day, plan selection, synthetic CT

## Abstract

**Introduction:**

In the Library‐of‐Plans (LoP) approach, correct plan selection is essential for delivering radiotherapy treatment accurately. However, poor image quality of the cone‐beam computed tomography (CBCT) may introduce inter‐observer variability and thereby hamper accurate plan selection. In this study, we investigated whether new techniques to improve the CBCT image quality and improve consistency in plan selection, affects the accuracy of LoP selection in cervical cancer patients.

**Materials and methods:**

CBCT images of 12 patients were used to investigate the inter‐observer variability of plan selection based on different CBCT image types. Six observers were asked to individually select a plan based on clinical X‐ray Volumetric Imaging (XVI) CBCT, iterative reconstructed CBCT (iCBCT) and synthetic CTs (sCT). Selections were performed before and after a consensus meeting with the entire group, in which guidelines were created. A scoring by all observers on the image quality and plan selection procedure was also included. For plan selection, Fleiss' kappa (κ) statistical test was used to determine the inter‐observer variability within one image type.

**Results:**

The agreement between observers was significantly higher on sCT compared to CBCT. The consensus meeting improved the duration and inter‐observer variability. In this manuscript, the guidelines attributed the overall results in the plan selection. Before the meeting, the gold standard was selected in 76% of the cases on XVI CBCT, 74% on iCBCT, and 76% on sCT. After the meeting, the gold standard was selected in 83% of the cases on XVI CBCT, 81% on iCBCT, and 90% on sCT.

**Conclusion:**

The use of sCTs can increase the agreement of plan selection among observers and the gold standard was indicated to be selected more often. It is important that clear guidelines for plan selection are implemented in order to benefit from the increased image quality, accurate selection, and decrease inter‐observer variability.

## 1 INTRODUCTION

The standard treatment for locally advanced cervical cancer is combination of chemotherapy, external beam radiotherapy, and brachytherapy.^1^ In radiotherapy, treatment sites with large daily variation in anatomy pose a challenge for radiation therapy technologists (RTTs) performing the daily treatments. For example, in the pelvic area day‐to‐day variations are present due to differences in bladder and rectum filling.^2^ This variation may cause large deformations of the cervix and uterus.^3^ Although drinking protocols aid in stabilizing bladder filling, resulting in less inter‐fraction motion, the day‐to‐day variation can still be substantial.^4^, ^5^

To be able to partly adapt the treatment plan to the daily anatomy, the concept of a Library of Plans (LoP), also known as Plan‐Of‐the‐Day (PotD), was introduced.^6^, ^7^, ^8^ LoP is nowadays widely used for multiple treatment sides in the clinic. In LoP, multiple treatment plans are made prior to the treatment series. The multiple plans consist of a full bladder plan, empty bladder plan, and several interpolated plans in‐between. The number of plans is dependent on the amount of clinical target volume (CTV) displacement between the full bladder computed tomography (CT) scan and the empty bladder CT scan.^9^ Prior to each treatment fraction, the treatment plan that is closest to the daily anatomy on the Cone‐Beam CT (CBCT) is selected out of the generated LoP.

Selecting the correct plan is essential for accurate radiotherapy, but may be hampered by inter‐observer variability. In cervical cancer LoP, previous studies have reported that 77% of the plans selected by different observers were in concordance with the defined gold standard plan, when selection was performed on CBCT using fiducial markers.^10^ The plan selection is, however, often challenged by poor visibility of the cervix on the CBCT, due for example, to air pockets in the rectum and bowel, resulting in streaking artefacts.

Poor image quality of the CBCT could increase the inter‐observer variability of plan selection among observers and decrease the accuracy of plan selection. New techniques for image reconstruction and synthesis may improve CBCT image quality and thereby have a beneficial influence on the LoP selection. For example, the iterative reconstructed CBCT (iCBCT) method is a technique in which the reconstruction is performed multiple times. Another method entails producing a synthetic CT (sCT) using a deep‐learning algorithm. The aim of this study was to evaluate the differences between the clinical CBCT image, iterative reconstructed CBCTs (iCBCT), and sCTs on the LoP selection for cervical cancer patients.

## 2 MATERIALS AND METHOD

### 2.1 Patient data

This study retrospectively included data of 12 cervical cancer patients that received radiotherapy treatment between April 2021 and March 2022 at Catharina Hospital Eindhoven. The age of the patients was between 29 and 84 years (average 47 years). This research was conducted on anonymized patient data and, according to Dutch law, this research was approved for the medical research law waiver (under “non‐WMO” legislation).

All patients were treated according to the EMBRACE II protocol.^1^ A drinking protocol was used to obtain a sufficiently filled bladder for the full bladder CT scan. Patients were asked to void  h before CT scan imaging and CBCT imaging at each radiotherapy treatment fraction, and to drink 250–400 mL of water. Planning CT (pCT) images were acquired with oral contrast for small bowel and bladder visualization. For each patient, full and empty bladder CT images were acquired on a Philips big bore CT (Philips, Eindhoven, Netherlands) with 120 kVP, 512 × 512 voxels, 3 mm slice thickness. Before each fraction, CBCT images were acquired on X‐ray Volumetric Imaging (XVI) v5.0.4 systems (Elekta AB, Stockholm, Sweden). For each patient, CBCT images of two fractions per patient were selected for the present study based on different levels of bladder and rectum filling.

### 2.2 Library of plans

Organs at risk (OAR) and target structures were contoured on full and empty bladder CT scans according to the EMBRACE II guidelines. The Clinical Target Volume (CTV) consisted of CTV_uterus, CTV_cervix, and CTV_vagina. These contours were used to generate a library of plans. The number of plans generated per patient ranged between two and five plans, which was dependent on the displacement of the CTV between the full and empty bladder scan. Hausdorff 95th percentile distance (HD95) between CTVs of the different plans was on average 15 mm and ranging from 8 to 26 mm.

### 2.3 Evaluated CBCT image types

#### 2.3.1 Clinical XVI CBCT

CBCT images were acquired and reconstructed on XVI v5.0.4 systems (Elekta AB, Stockholm, Sweden), which included filtering of the projections and a FDK reconstruction.^11^ Image settings were: medium Field Of View (FOV) with a F1 bowtie filter, 120 kV, 64 mA/frame, 40 ms/frame, 660 projections in a full rotation arc of 360 degrees.

#### 2.3.2 Iterative reconstructed CBCT

The iCBCT was reconstructed with Framework for Reconstruction and Simulation of Conebeam images V21.1.0 (FRESCO) (Elekta AB). The reconstruction pipeline of FRESCO is the XVI v5.0 pipeline with additional algorithms such as lag correction, improved beam hardening correction, glare correction, and a Monte Carlo scatter correction.^12^, ^13^, ^14^ Simulation and reconstruction steps were iteratively performed five times.

#### 2.3.3 Synthetic CT

XVI CBCT images were used for sCT generation with Advanced Medical Image Registration Engine research V3.37.0 (ADMIRE) (Elekta AB). The ADMIRE Model used was EKT CBCT‐sCT M + F Pelvis v2.0. The model was trained on paired CBCT and pCT female and male pelvic data from different hospitals by Elekta. The model consist of a 2D cycle‐GAN to generate sCTs.^15^

### 2.4 Image quality

To assess a quantitative measure of the image quality, Peak‐Signal‐to‐Noise Ratio (PSNR) was calculated within the bladder contour, which was delineated on XVI CBCT, iCBCT and sCT. These delineations were evaluated and, if necessary, adapted by an experienced radiation oncologist. To determine the anatomical correctness of the images, the average surface distance (ASD) and 95^th^ percentile Hausdorff distance (HD95) between the bladder contour on the XVI CBCT and bladder contour on the iCBCT or sCT was calculated.^16^

### 2.5 Plan selection and radiological scoring

**FIGURE 1:**
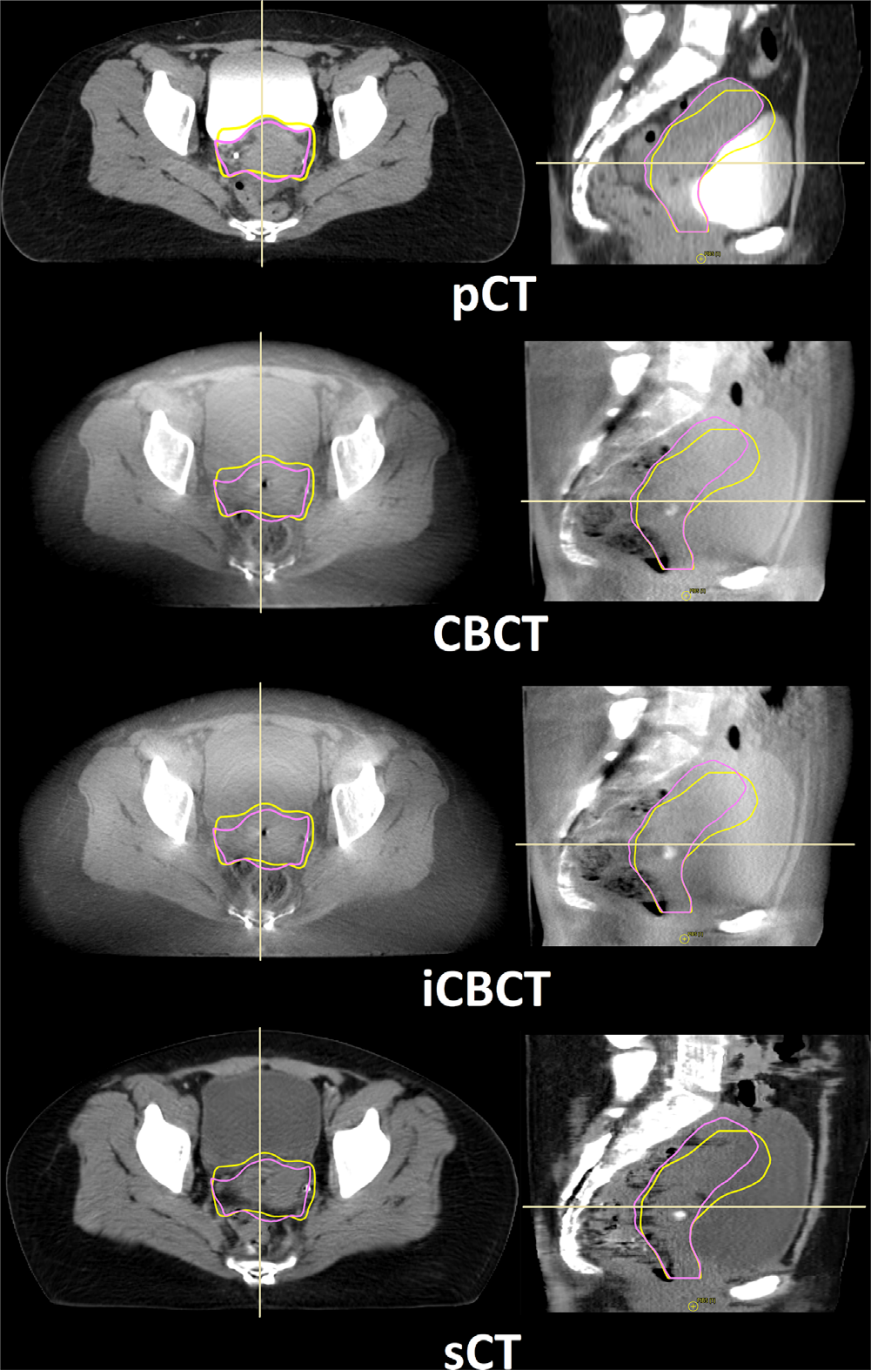
A typical example of the different types of images from one patient. From top to bottom, planning CT, XVI CBCT, iterative reconstructed CBCT generated with FRESCO and synthetic CT image generated from XVI CBCT with ADMIRE. Left to right in each row, transversal view and sagittal view. The yellow line is the CTV for the empty bladder plan and the pink line is the CTV for the full bladder plan.

Six observers; four RTTs, one radiation oncologist and one medical physicist, were asked to select a treatment plan from the library of plans for the XVI CBCT, iCBCT, and sCT images. The observers were asked to perform the plan selection according to the current clinical practice, with the exception that plan selection needed to be performed individually. A total of 72 images were used for plan selection, consisting of three image types per fraction and two fractions per patient. The order of images was determined randomly for each observer. The image set included a XVI CBCT or iCBCT or sCT image and a full bladder pCT image as reference with CTV, Planning Target Volume (PTV), and bladder contours (Figure 1).

The median selected plan per fraction and image type was considered as the gold standard. The median rather than the average plan was used, since the data had a non‐normal distribution. If the median was between two plans, the selection by the radiation oncologist was set as the gold standard. The radiation oncologist was selected to have more weight in this case due to his/her clinical and anatomical expertise as well as responsibility for the intended treatment. These gold standard plans were used to measure the distance between selected plans and the gold standard in the number of plans as well as the HD95 distance. The HD95 distance was used instead of the ASD since the HD95 is more sensitive to the movable cranial part of the CTV.

Next to the plan selection, a scoring by all the observers on the image applicability was included. Questions were asked about how observers selected a specific plan and image quality. The questions about the plan selection procedure related to the user's degree of confidence and time needed for plan selection, to be able to mimic the clinical plan selection procedure. The options for confidence were: “no hesitation between plans”, “hesitation between 2 plans”, “hesitation between 3 or more plans”. The options for time needed for plan selection were: “fast plan selection”, “longer time needed”, “I would have consulted a radiation oncologist or a medical physicist”. In the questionnaire of the radiation oncologist and medical physicist, the latter option was changed to “the RTT should have consulted a radiation oncologist or a medical physicist.” To obtain information about the image quality, observers were asked to grade image quality, number of artefacts and the visibility of bladder, cervix and rectum. A scale of 1−5 was used, with one being the best and five the worst image quality. The artefacts were graded on a scale consisting of the following options: “few artefacts”, “several artefacts, but not hampering plan selection”, “many artefacts with plan selection still possible”, and “too many artefacts to allow for plan selection”. The options for organ visibility were on a scale of 1−5: “boundary of organ is visible”, “overall organ is visible”, “organ is partly visible”, “organ is barely visible”, “organ is not visible”.

After the first scoring and plan selection by the six observers, a consensus meeting about the differences in plan selection led to selection guidelines. The guidelines concerned the plan selection procedure, including cases where the CBCT CTV was identified as located between two library CTVs. If this was the case, the plan made for the larger bladder size of those two plans should be selected in order to lower the small bowel dose. Additionally, selecting the larger bladder size slightly anticipates on additional bladder filling during the treatment fraction. Two months after the consensus meeting, a second round of plan selection was performed, in which plan selection was performed according to the consensus guidelines. In this second round, one fraction for each patient was included that contained all three reconstruction (XVI CBCT, iCBCT, and sCT) types to check if the consensus meeting had a similar effect on each of the image types.

### 2.6 Statistical analysis

For plan selection, Fleiss' kappa (κ) was run to determine the inter‐observer variability within one image type.^17^ κ = −1 indicates observed dis‐agreement, κ = 0 indicates that agreement was no better than chance, κ = 1 indicates perfect agreement between observers. A κ between 0.41 and 0.60 indicates a moderate agreement and a κ between 0.61 and 0.80 indicates a substantial agreement.^18^ The kappa value was calculated for each fraction and image type. Therefore, each image type consists of a distribution of kappa values, which results in a confidence interval per image type. To compare each pair of two image types mutually, the kappa values were tested on significance with a two tailed z‐test.^19^ This way, the z‐test was used three times. Therefore, the significance level of the z‐test was corrected for multiple testing with the Bonferroni correction to *p* ≤ 0.017.

For the questions about plan selection procedure and image scoring, a Wilcoxon Signed‐Rank (WSR) test was used to test whether the image types were statistically different.^20^ Prior to the WSR test, however, a Friedman test was used to determine whether the WSR test was allowed, which was necessary as more than two groups were compared.^21^ The Friedman test, which is a non‐parametric alternative to a repeated measures ANOVA, was used with a significance level of *p* ≤ 0.05. If the Friedman test showed an overall significant difference, a WSR test was used to compare each pair of two image types mutually. Because the WSR test was hereby utilized three times, its significance level was corrected for multiple testing (*p* ≤ 0.017). For simplicity, if Friedman was significant, only the *p* values of the WSR are shown.

## 3 RESULTS

### 3.1 Image quality

A typical example of the different types of images from one patient is illustrated in Figure 1. The iCBCT was sharper compared to XVI CBCT. There was more contrast between bladder and CTV in sCT compared to XVI CBCT and iCBCT. However, the rectal structure was less visible in the sagittal view of the sCT due to discontinuous boundaries of the rectum. This type of image degradation was caused by the generation of sCTs, which was done per slice.

**FIGURE 2:**
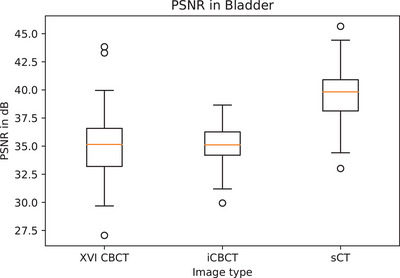
Boxplot of quantitative image quality evaluation by PSNR of three image types.

**FIGURE 3:**
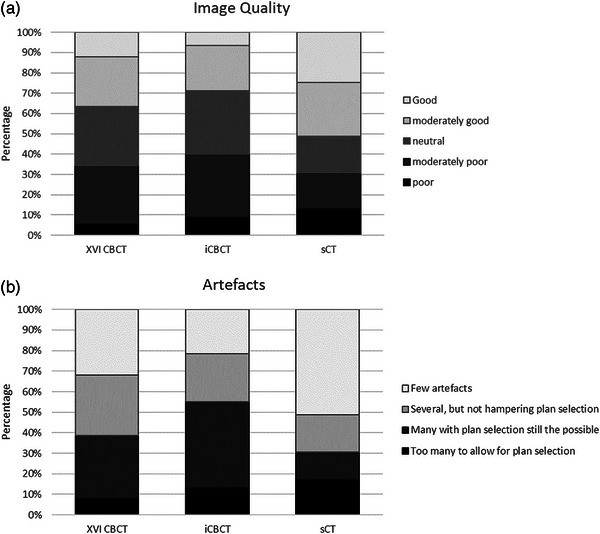
Stacked column chart of the image scoring. (a) Image quality. (b) Artefacts.

**FIGURE 4:**
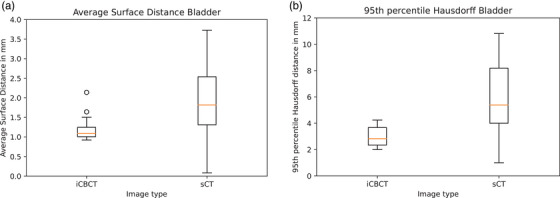
Boxplots of anatomical image evaluation. (a) Bladder anatomy evaluation of iCBCT and sCT images, the average surface distance between bladder contour on XVI CBCT and iCBCT or sCT. (b) HD95 between bladder contour on XVI CBCT and iCBCT or sCT.

The image quality in terms of PSNR was significantly higher in sCT, compared to iCBCT (*p* < 0.001) and XVI CBCT (*p* < 0.001), median 40 dB [range: 33–46 dB] compared to 35 dB [range: 27−44 dB] and 35 dB [range: 30−39 dB], respectively (Figure 2). In the image quality scoring by the observers, sCT scored higher compared to XVI CBCT (*p* = 0.006) and iCBCT (*p* = 0.004). More sCT images were scored as “good” and “moderately good” 38% compared to 23% on XVI CBCT and 14% on iCBCT (Figure 3a). However, fewer XVI CBCT images were scored as “poor” 7% compared to 13% on iCBCT and 14% on sCT. On the artefacts scoring by the observers, sCT scored highest on “few artefacts” 51% compared to 32% XVI CBCT (*p* < 0.001) and 21% iCBCT (*p* < 0.001) (Figure 3b). On the anatomical evaluation of the bladder, iCBCT scored significantly better on ASD between XVI CBCT and iCBCT compared to ASD between XVI CBCT and sCT (*p* = 0.002), median 1.1 mm [range: 0.9–2.6 mm] and 1.8 mm [range: 0.1–3.7 mm], respectively (Figure 4).

### 3.2 Plan selection

**FIGURE 5:**
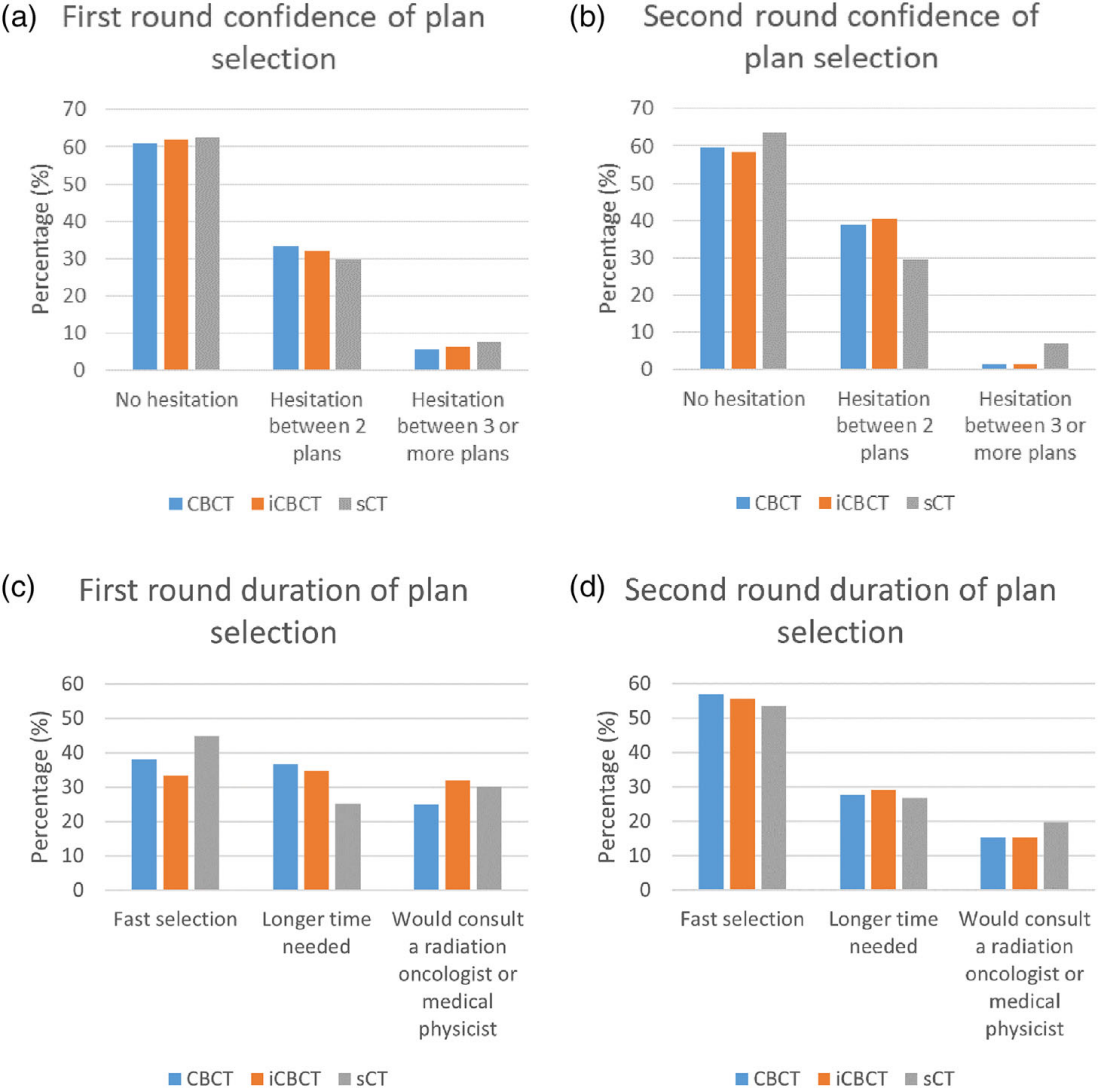
Bar plot confidence and duration of plan selection. Top row, the confidence of plan selection in the first and second round. On the bottom row, the level of duration of plan selection in the first and second round.

The level of confidence in plan selection and time needed to select a plan was evaluated. In the first round, observers scored “no hesitation between plans” in 61% of the cases on XVI CBCT image, 62% on iCBCT image, and 63% on sCT image (Figure 5). In the second round the level of confidence was not improved compared to the first round. Observers scored “no hesitation between plans” in 60% of the cases on XVI CBCT image, 58% on iCBCT image, and 63% on sCT image. In the first round, fast plan selection was selected in 38% of cases on CBCT image, 33% on iCBCT, and 45% on sCT. “Would consult a radiation oncologist or medical physicist” was selected in 25% of the cases on CBCT, 15% on iCBCT, and 32% on sCT. In the second round the duration of plan selection was improved significantly compared to the first round in XVI CBCT (*p* = 0.050), iCBCT (*p* < 0.001), and sCT (*p* = 0.008). “Fast plan selection” was selected in 57% of cases on XVI CBCT, 56% on iCBCT, and 54% on sCT. “Would consult a radiation oncologist or medical physicist” was selected in 15% of the cases on CBCT, 15% on iCBCT, and 20% on sCT.

### 3.3 Agreement between observers

**FIGURE 6:**
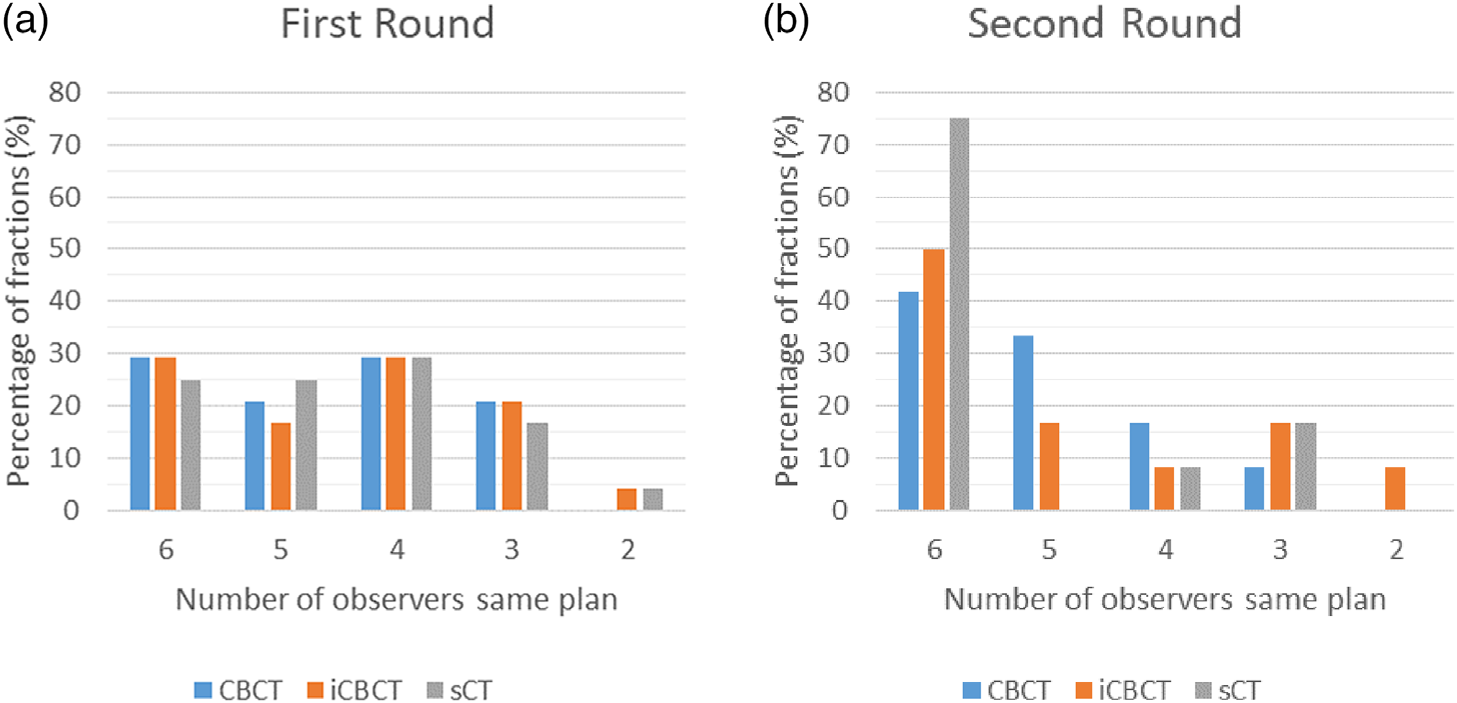
Barplot of plan selection agreement. The number of observers which selected the same plan. The colors indicate the three different image types, CBCT, iCBCT, and sCT.

The level of agreement between observers on plan selection was evaluated. The maximum number of observers that selected the same plan varied per fraction and image type (Figure 6a). Full agreement was reached in: 29% of the fractions based on the XVI CBCT, 29% on iCBCT, and 25% on sCT. In the second round, this improved to 42% by selection based on CBCT, 50% on iCBCT, and 75% on the sCT.

In the first round, Fleiss’ kappa, the measurement for inter‐observer variability, showed that there was moderate agreement between observers for all three image types (Table 1). In the second round, the κ was increased in all three image types; however, the amount of increase was different per image type. The κ was significantly higher in sCT compared to CBCT (p = 0.007) and iCBCT (p = 0.015). In the sCT, κ indicated that there was substantial agreement between observers. In XVI CBCT and iCBCT, still moderate agreement between observers selected plans was observed.

**TABLE 1:** Inter‐observer variability of the three different image types, expressed in Kappa values.

Image type	XVI CBCT	iCBCT	sCT
First round kappa [κ (95% CI)]	0.47 (0.40–0.52)	0.52 (0.45 to 0.58)	0.50 (0.43–0.56)
Second round kappa [ κ (95% CI)]	0.58 (0.48–0.68)	0.60 (0.50–0.70)	0.77 (0.67–0.89)

### 3.4 Distance to gold standard

**FIGURE 7:**
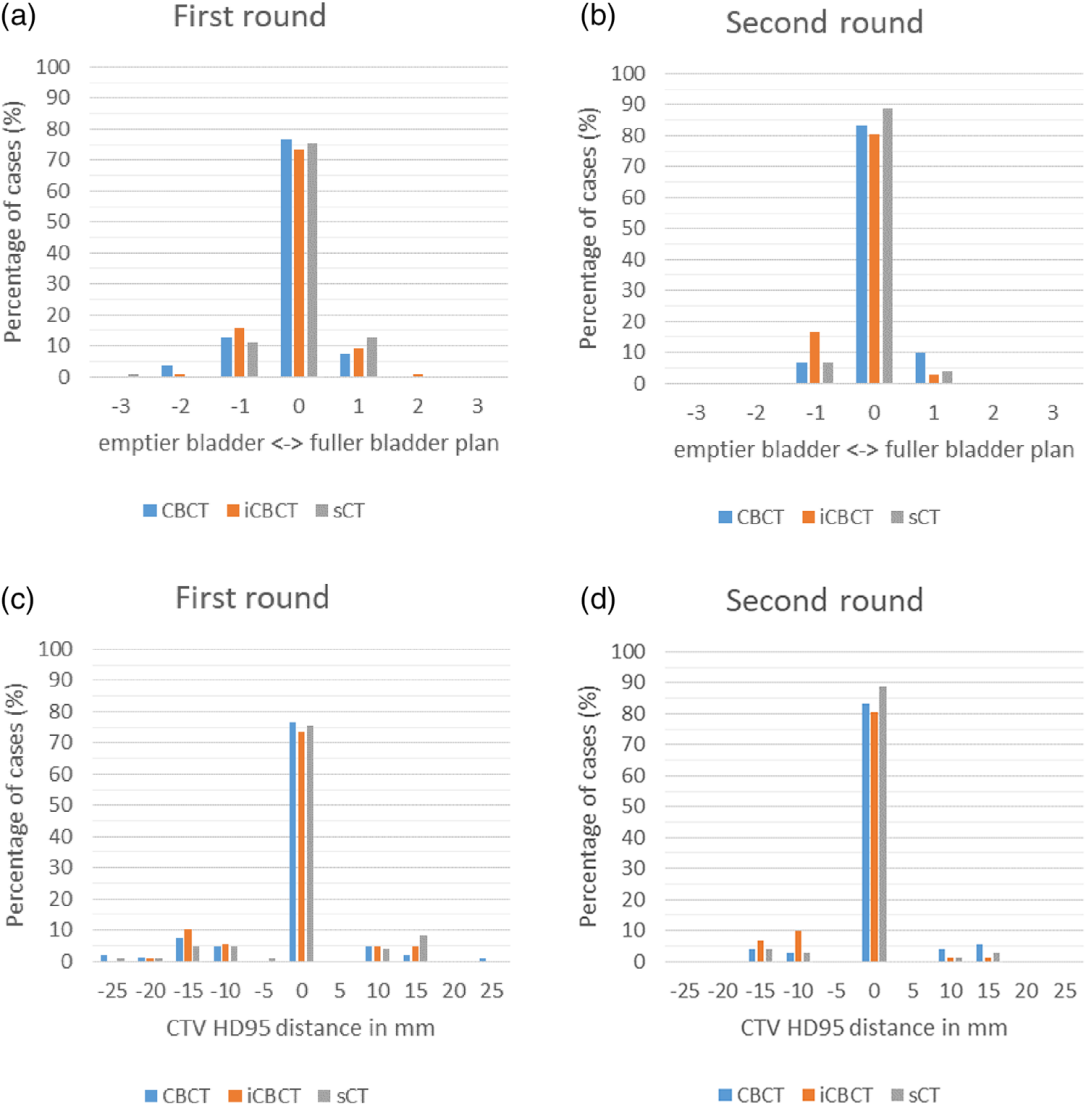
The difference between selected plan and gold standard plan. On the top row, the percentage of cases the gold standard was selected, a larger or smaller plan compared to the gold standard plan. On the bottom row, 5 mm bin size histogram of the selected plans categorized on HD95 between CTV selected plan and gold standard plan. Negative values indicate a plan which was based on an more empty bladder than gold standard plan. Value of zero indicate that gold standard plan was selected. A positive value indicates a plan based on a fuller bladder than gold standard plan. The colors indicate the three different image types, XVI CBCT, iCBCT, and sCT.

In the first round, the gold standard was selected in 76% of the cases on XVI CBCT, 74% of the cases on iCBCT, and 76% of the cases in sCT (Figure 7). Although there was no significant difference between the first and second round distribution of preferred image type, gold standard plans were selected more often in the second round compared to the first round. Percentage of cases a gold standard was selected was 83% on XVI CBCT, 81% on iCBCT, and 90% on sCT in the second round.

The selected plans were evaluated on amount of difference compared to the gold standard plan, measured in number of plans and CTV HD95 distance. One plan removed from the gold standard plan, in other words, the plan made for a smaller or larger bladder than the gold standard plan, was selected in 20% on XVI CBCT, 25% on iCBCT, and 24% of cases on sCT. If a smaller or larger plan was selected, the median CTV HD95 increased by 13 mm [range: 8–26 mm] in CBCT, 13 mm [range: 8–17 mm] in iCBCT and 13 mm [range: 7–26 mm] in sCT. The plan removed more than once from the gold standard was selected in 3% on XVI CBCT, 1% on iCBCT, and 1% on sCT. In the second round, a maximum of one plan for a smaller or larger bladder than the gold standard was selected. If a smaller or larger plan was selected, the median CTV HD95 increase by 13 mm [range: 8–17 mm] in CBCT, 10 mm [range: 8–17 mm] in iCBCT, and 13 mm [range: 8–13 mm] in sCT.

## 4 DISCUSSION

In this study, a significantly higher agreement between observers was found in Library of Plan selection based on sCT compared to XVI CBCT and iCBCT. Additionally, a consensus meeting resulted in higher agreement between observers due to the smaller distance achieved between the selected plan and gold standard plan. Before the consensus meeting, the percentage of plans in concordance with gold standard was comparable to current literature.^10^, ^22^ The percentage of plans in concordance with gold standard was higher in the second round.

In the first round, all image types showed moderate agreement between the plan selections of the observers. In the second round sCT showed strong agreement while CBCT and iCBCT showed moderate agreement. The improvement of agreement was greatest for sCT than iCBCT or CBCT. The improvement in the second round could be due to the implementation of guidelines, as demonstrated in de Jong et al. with plan selection on CBCT images.^22^ On the other hand, iCBCT and sCT were new image types to the observers, so a learning‐curve for plan selection on those new image types could cause additional improvement. The results of the questionnaire support the improvement in agreement, showing that fast plan selection was possible more frequently, and that consulting a radiation oncologist or medical physicist for guidance was needed less often. Faster plan selection and less consulting of radiation oncologist or medical physicist results in shorter treatment times in the clinic.

The inter‐observer variability was significantly lower if plan selection was based on sCT in the second round. However, there was no significant difference between image types in plan and CTV HD95 distance to gold standard, only an indication that the gold standard was selected more often in sCT. This difference in significance of inter‐observer variability and comparison to gold standard can be explained due to the requirement of a gold standard in plan distance to gold standard. For inter‐observer variability no gold standard was required. For the distance to gold standard plan, median selected plan per patient and image type was considered as the gold standard. The real ground truth cannot be determined at the precise time of treatment as surrogates for the precise location of the target are evaluated. More observers could improve the accuracy of the gold standard approximation. On the other hand, this could increase inter‐observer variability and thereby reduce consistency in clinical plan selection.

If a smaller or larger bladder plan than the gold standard was selected, on average the CTV HD95 was increased by 13 mm. These displacement differences are clinically relevant, since the EMBRACE II protocol prescribes cervix PTV margins of 5−10 mm^1^. The radiation treatment of cervical patients was divided in 25 fractions. Since, after the consensus meeting, in only 10% of the cases another plan was selected on sCT images, this suggests that only for two or three fractions another plan would be selected. However, the consistency in plan selection was different per patient. This difference could have an influence on the received treatment dose and side effects. In further research, the impact of plan selection on the radiotherapy treatment of the patient needs to be investigated, in terms of dose to the target volumes and organs at risk.

sCT scored significantly better in plan selection agreement, image quality and fewer artefacts compared to CBCT and iCBCT. Nevertheless, the sCT images could be even further improved in sagittal view, for example, with the 2.5D or 3D training of sCT image generators.^23^, ^24^, ^25^ Since plan selection was in our experience mostly done on the sagittal view, the image quality of the sagittal slices is important. The location of artefacts is also important, since in our experience the plan was selected on small features, such as a slight fat boundary between the organs, to locate the CTV. Next to the influence of artefacts on the plan selection, artefacts could also have an influence on other radiotherapy‐related features such as dose calculations.

The advantage of sCT in the clinical workflow is that the sCT generation time is shorter compared to iCBCT reconstruction (approximately half a minute for the sCT generation compared to 5 min for the iCBCT reconstruction). XVI CBCT reconstruction takes approximately 35 s for pelvic area scans. However, the XVI CBCT reconstruction can occur during acquisition of the projection images, which would also be possible for the iCBCT reconstruction. The sCT generation has to occur after the XVI CBCT reconstruction. The advantage of CBCT and iCBCT, however, is that the geometry and anatomy is accurate. sCT, on the other hand, had a significant difference between bladder contour ASD on CBCT and sCT. The ASD was less than two times the ASD of deep‐learning segmentations compared to manual delineations of bladder in female pelvis.^26^, ^27^, ^28^ This variation can be due to the performance of anatomical preservation of the deep‐learning network.^16^, ^29^ If the anatomical preservation is low, it could influence plan selection.

A limitation of this study was the included number of patients. Since several image types were evaluated per patient, the number of patients was limited to twelve patients. The agreement between observers in plan selection based on sCT was significantly higher compared to XVI CBCT and iCBCT. This result is expected to be consistent if a larger number of patients would be included.

## 5 CONCLUSION

In conclusion, the use of sCTs can improve the agreement of plan selection among observers. Moreover, image quality improvement increases the accuracy of plan selection and the agreement between observers notably after implementing guidelines about plan selection. Therefore, image quality as well as training in plan selection is important for improving the accuracy and consistency of LoP.

## AUTHOR CONTRIBUTIONS

All authors contributed to the conception of the work, analysis and interpretation of data. All authors contributed to and approved the final version of the manuscript. All authors agreed to be accountable for the work in ensuring that questions related to the accuracy or integrity of the manuscript are appropriately investigated and resolved.

## CONFLICT OF INTEREST STATEMENT

The authors declare no conflicts of interest.
